# Awareness of Autism in Primary School Teachers

**DOI:** 10.1155/2013/961595

**Published:** 2013-11-30

**Authors:** Muhammad Mustafa Arif, Ayesha Niazy, Bilal Hassan, Farah Ahmed

**Affiliations:** Department of Community Health Sciences, Ziauddin University, 4/B, Shahrah-e-Ghalib, Block 6, Clifton, Karachi 75600, Pakistan

## Abstract

*Objective*. To assess the knowledge and perception of primary school teachers regarding autism in private and public schools of Karachi, Pakistan. *Methods*. A cross-sectional survey was conducted on primary school teachers in different districts of Karachi. A sample size of 170 teachers was selected by purposive sampling. Primary data was collected using self-administered questionnaires. These questions assessed the teacher's knowledge and perception of Autism. Data was entered on SPSS version 20. Frequencies and percentages were taken out for categorical variables. *Results*. Of the total 170 teachers, 85 were from the Private and 85 from Public sector schools. 55% (*n* = 94) of the teachers knew about Autism through the media and only 9% (*n* = 15) had formal training through workshops on Autism. 62% (*n* = 105) of the teachers were of the opinion that Autism is treatable. Majority of the teachers (57%) said that proper training is required for teaching autistic children. *Conclusion*. The knowledge related to Autism in our existing sample has mostly come from the media. Although we cannot undermine the role of media, there is a need to give formal training to teachers regarding the differentiating features of Autism, which in turn will aid in early diagnosis of the disease.

## 1. Introduction

Autism is a disorder of neural development, characterized by impaired social interaction, communication, and by restricted, repetitive behavior [1]. This condition begins at birth or within the first two-and-a-half years of life. The children affected are perfectly normal in appearance, but they spend their time engaged in puzzling and disturbing behaviors which are markedly different from those of typical children [2].

The cause of autism is not known [3, 4]. Studies suggest that there is a strong genetic basis, but it cannot be traced to a Mendelian (single-gene) mutation or to a single chromosome abnormality [5, 6]. Risk factors for autism include gender, males are three to four times more likely than females to get autism, family history, parents' age, and other disorders such as fragile **x** syndrome, tuberous sclerosis, Tourette's syndrome, and epilepsy [7]. Apart from these, the use of drugs during pregnancy has also been identified as a risk factor. This was evident by a retrospective case study, which showed that maternal valproic acid used during pregnancy caused autism in the newborn [8]. Studies have even suggested the association of congenital rubella with autism [9, 10]. Another relatively frequent medical condition is cerebral palsy [11], in which the rates of children getting autism are high.

People with autism can have very different features or symptoms; thus, health care providers think of autism as a spectrum disorder [3]. Some children with autism make no eye contact and seem aloof, while others may show intermittent engagement with the environment and may make inconsistent eye contact, smile, and hug. Children may also exhibit varying verbal abilities, ranging from being nonverbal to having advanced speech.

Intellectual functioning can vary from mental retardation to superior intellectual functioning in selected areas. Some children with autism showed typical development in certain skills and even showed strengths in specific areas, such as puzzles, art, and music. However, in general, an Autistic child is often withdrawn and spends hours in solitary play [12].

Several screening tools have been developed to aid in early detection of children with autism Spectrum Disorder. The Checklist for autism in Toddlers (CHAT) is a screening tool designed for 18-month-old children in primary care settings. The Modified Checklist for autism in Toddlers (M-CHAT) is a twenty-three-item parent questionnaire for screening children between 16 and 30 months of age to assess their risk for autism spectrum disorder. The pervasive developmental disorder screening test (PDDST) is a parent completed survey that targets children from birth to three years of age [12].

Studies in Asia, Europe, and North America have identified individuals with an autism spectrum disorder with an approximate prevalence of 0.6%–1% of the newborn population [13]. A study conducted in the 50 states in the USA checked the differences amongst them, in terms of identification of autism spectrum disorders [14]. It was concluded that states with better educational and healthcare expenditures were more apt in the diagnosis of autism.

According to Pakistan's country report on autism, the prevalence of the disease in children is reported to be approximately 1 in 120 [15]. The actual reported prevalence of autism is much lower in Pakistan than that in the western world, due to the lack of awareness about it. The general public is unaware of the severity of the disease and how to diagnose it. The object of this study is to determine the level of awareness of autism in Primary school teachers. The results of this study will help schools and organizations increase the awareness levels related to autism, so it can be diagnosed at an early age and intervention can be started accordingly.

## 2. Materials and Methods

This was a cross-sectional study conducted on Primary School Teachers in different districts of Karachi.

Sample size was calculated to be 160 by keeping confidence interval at 95%, precision at 5%, and prevalence at 1%. Wastage was added and the sample size was inflated to increase the validity of the study. A total of 170 teachers were selected by simple random sampling (SRS). Only the teachers teaching students form grades 1 to 5, with at least three years of teaching experience, were included in the study. Primary data was collected from teachers through questionnaires which assessed the teacher's knowledge of autism.

The questionnaire was designed to assess different aspects of their knowledge such as diagnosis, symptoms, and treatment. Questions regarding symptomalogy of autism were based on the diagnostic criteria by the American Psychiatric Association Diagnostic and Statistical Manual for Mental Disorders, Fourth Edition. After the questionnaires were filled, the data was analyzed using SPSS 20. The teachers were divided into two categories (i.e., private and nonprivate). Frequencies and percentages were taken out for each category. Chi-square test was applied to find out differences between the two categories and *P*  value less than 0.05 was taken as significant.

An average value of the number of correct responses amongst the teachers from both sectors was taken out. These values were then compared using Chi-square test and Fisher exact test, where applicable.

## 3. Results

**Figure 1:**
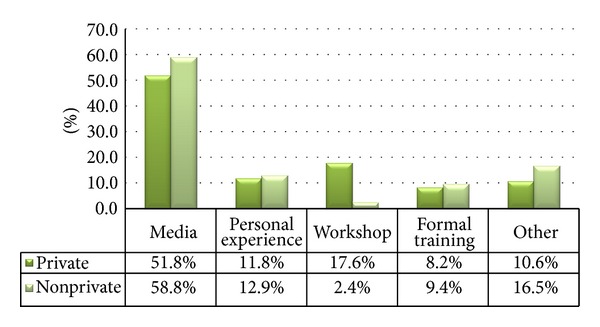
Source of awareness of autism according to institution type.

Data from 170 teachers from private and nonprivate sectors was analyzed. It was determined that 55% (*n* = 94) of the sample had acquired their knowledge of autism through media (see Figure 1). Only 10% (*n* = 17) of the sample had attended formal training whereas 8.8% (*n* = 15) had attended a workshop regarding autism. The source of information regarding autism in the rest of the sample was either from personal experiences or other sources.

The education level of both the private and nonprivate samples was also compared. 33% (*n* = 28) of the sample in the nonprivate sector had an M.S. degree, whereas the remaining had a B.S. degree. Comparing this to the education level of the teachers teaching in the private sector, it was determined that 31% (*n* = 26) had M.S. degree, 60% (*n* = 51) had a B.S. degree, and 9% (*n* = 8) just had a college education.

The questionnaire had 16 questions regarding basic knowledge and perception of autism. The first 13 questions tested the teacher's basic knowledge about autism (see Table 1). Only 22% (*n* = 19) of the teachers in the Private sector and 28% (*n* = 24) of the teachers in the nonprivate sector were aware that autism is an inherited disorder. When inquiring about what type of disorder autism is, 48% (*n* = 41) of the teachers in the Private sector and 46% (*n* = 39) of the teachers in the nonprivate sector recognized the fact that autism is both a learning and a mental disorder.

On comparing responses regarding the knowledge of autism in both categories, we found that there was no statistical significance, except in two categories: communication skills of an Autistic child (*P*  value = 0.002) and emotional temperament of an Autistic child (*P*  value = 0.002) (see Table 1). Regarding the communication skills of an Autistic child, we saw that teachers in the public sector were better aware that an Autistic child has poor communication skills and cannot express himself. When we analyzed the results of emotional status of an Autistic child, we found out that teachers in the private sector were better aware of autism and recognized the fact that an autistic child throws frequent bouts of rage.

**Figure 2:**
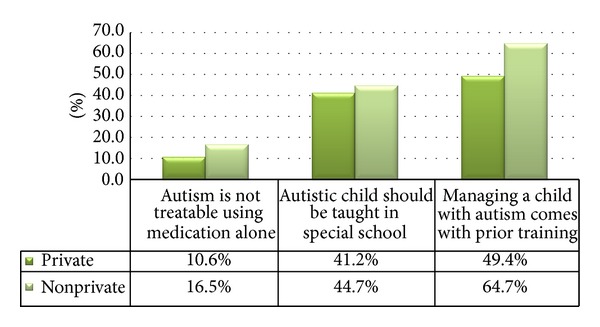
Perception of autism according to institution type.

On investigating the perception of autism in both categories (see Figure 2), we found out that *P* values were significant when teachers were questioned regarding the treatment of autism using, solely, medication (*P* value = 0.005). Participants in the nonprivate sector had a better perception of the treatment of autism and were aware that it is a condition that is not managed by medication alone, but a combination of medication and different behavioral and cognitive therapies. All the other responses regarding perception of autism had insignificant *P*  values.

We then compared the correct responses in both categories (see Table 2). Responses from the 16 questions on knowledge and perception were combined into two categories (i.e., right and wrong) (see Table 2). In the private sector, average no. of correct answers by a participant was 6.85 with a standard deviation of 2.89. This was considerably smaller when compared to the nonprivate sector, where average no. of correct answer per subject was 7.88 with a standard deviation of 3.23. The knowledge and perception scores were then compared using independent sample test which showed significant (*P*  value = 0.029) difference. Thus, we concluded that the teachers in the nonprivate sector had better knowledge and perception regarding autism when compared with their counterparts in the private sector.

## 4. Discussion

Autism is currently diagnosed in approximately 1 out of every 150 children [13]. With increasing prevalence of this condition, more awareness is needed amongst the population to diagnose it at an early age so that therapy can be initiated.

A study conducted in Singapore [16], amongst 503 preschool teachers, displayed the deficiency of teachers regarding knowledge, attitudes, and practices on childhood developmental and behavioral disorders. Similarly, in our study, we saw that, out of all the responses on knowledge, only 47.9% of the responses were correct. This clearly displayed the fact that majority of the population is not aware of the basic facts about autism.

Diagnosis of autism at an early age is highly beneficial in the intellectual development of the child. Early diagnosis of the disease enables initiation of appropriate therapy in children, aiding in their development. Teachers play a vital role in interaction with a child during the early years of life. Their ability to pick out Autistic children in the classroom will be beneficial on the long term.

Management of autism includes a combination of medication plus behavioral and cognitive therapies [17]. A clinical trial conducted by Ghanizadeh and Moghimi-Sarani [18] concluded that risperidone and acetylcysteine, in combination, decreased irritability in children with autism but had no effect on the core symptoms of autism. Similarly, in a systemic review of medications commonly used to treat autism, it was seen that although medications are commonly used for treatment of autism, there is little evidence of effectiveness of these treatments [19].

A catalogue published by the Illinois Weselan University [20] in 2005 greatly emphasizes the role of teacher training in management of autism Spectrum Disorders in infancy. In our study, even though 57% (*n* = 91) of the teachers realized the importance of getting prior training when dealing with children with autism, only 8% (*n* = 7) of teachers in the private sector and 9% (*n* = 8) of the teachers in the nonprivate sector had attended formal training on autism. This clearly displays the lack of training opportunities regarding autism for teachers.

The Ministry of Education, British Columbia [21] published a resource book for teachers dealing with autism in 2000. Multiple tools were given to help teachers when facing Autistic children. When the tool was used by teachers on Autistic children, they showed marked intellectual improvement and coped up well with the school work. A few of the kids also started attending a normal school later. This again emphasizes the fact that through proper training of teachers we can greatly improve the quality of education of autistic children and improve their intellectual ability.

Eldevik et al. [22], in 2012, reported favorable outcomes in children undergoing behavioral interventions in mainstream preschool settings compared to children who were receiving normal treatments. He reported that students in preschool settings had better IQ scores and better adaptive techniques.

## 5. Conclusion

Our research clearly elaborates the lack of awareness amongst teachers regarding autism. We suggest that schools implement proper training programs for teachers to train them in diagnosing Autistic children and then teach them accordingly.

## Figures and Tables

**Table 1:** Percentage of correct responses according to the type of institution.

	Private		Nonprivate		*P* value
	*n*	%	*n*	%	
Autism is an inherited disorder	19	22	24	28	.585
Autism is a learning and mental disorder	41	48	39	46	.954
Signs of Autism show between 0 and 3 years	30	35.3	35	41.2	.104
An autistic child is not social	47	55	57	67	.255
An autistic child has poor communication skills and cannot express himself	45	53	64	75.3	.002
Verbally, an autistic child will have a hard time phrasing a sentence	32	38	51	60	.012
Nonverbally, an autistic child does repetitive gestures to express himself	34	40	26	31	.512
The attention span of an autistic child is deficient	59	69	58	68	.443
General interests of an autistic child are restricted	37	44	43	51	.401
An autistic child maintains minimal eye contact with others	58	68	69	81	.112
General eating habits of an autistic child are normal	22	26	22	26	.081
An autistic child is resistant to change	42	49	53	62	.366
An autistic child throws frequent bouts of anger	45	53	28	33	.002

**Table 2:** Table comparing correct responses among private and nonprivate teachers.

	Total no. of questions (total no. of responses)	Total no. of correct responses *n* (%)	Average no. of correct responses per subject (mean ± SD)	Median no. of correct responses	*P* Value
Comparison of knowledge					
Private	13 (1105)	496 (44.9%)	5.84 ± 2.75	6	0.058
Non-Private	13 (1105)	563 (51%)	6.62 ± 2.63	7	
**Total**	**13 (2210)**	**1059 (47.9%)**	**6.23 ± 2.71**	**6.5**	

Comparison of perception					
Private	3 (255)	86 (33.7%)	1.01 ± 0.764	1	0.058
Non-Private	3 (255)	107 (42%)	1.26 ± 0.915	1	
**Total**	**6 (510)**	**193 (37.8%)**	**1.14 ± 0.849**	**1**	

Comparison of knowledge and perception combined					
Private	16 (1360)	582 (42.8%)	6.85 ± 2.89	7	0.029
Non-Private	16 (1360)	670 (49.3%)	7.88 ± 3.23	8	
**Total**	**16 (2720)**	**1252 (46%)**	**7.36 ± 3.10**	**7**	
